# An association between environmental factors and the IVS4+44C>A polymorphism of the *DMT1* gene in age-related macular degeneration

**DOI:** 10.1007/s00417-012-1966-z

**Published:** 2012-02-29

**Authors:** Daniel Wysokinski, Malgorzata Zaras, Mariola Dorecka, Maja Waszczyk, Jerzy Szaflik, Janusz Blasiak, Jacek P. Szaflik

**Affiliations:** 1 Department of Molecular Genetics, University of Lodz, Pomorska 141/143, 90-236 Lodz, Poland; 2 Department of Ophthalmology, Medical University of Warsaw and Samodzielny Publiczny Kliniczny Szpital Okulistyczny, Sierakowskiego 13, 03-709 Warsaw, Poland; 3 Department of Ophthalmology, Medical University of Silesia, Ceglana 35, 40-514 Katowice, Poland

**Keywords:** AMD, Age-related macular degeneration, Oxidative stress, Iron homeostasis, Gene polymorphism, Divalent metal transporter 1, DMT1

## Abstract

**Background:**

Age-related macular degeneration (AMD) is an ocular disease affecting macula — the central part of the retina, resulting in the degeneration of photoreceptors and retinal epithelium and causing severe central vision impairment. The pathophysiology of the disease is not completely known, but a significant role is attributed to genetic factors. The contribution of oxidative stress in AMD as a trigger of the degenerative process is well-established. Iron ions may act as a source of reactive oxygen species; therefore, maintaining iron homeostasis is important for redox balance in the organism. Diversity in iron homeostasis genes may counterpart in unbalanced redox state, and thus be involved in AMD pathophysiology.

**Methods:**

In this work, we searched for an association between some single nucleotide polymorphisms in the divalent metal transporter 1 (DMT1) gene intronic IVS4+44C>A (rs224589) and 3’-UTR c.2044T>C (rs2285230) and environmental factors and AMD. Genotyping was performed using the PCR-RFLP method. DNA was obtained from 436 AMD patients and 168 controls.

**Results:**

We did not find any association between the genotypes of the two polymorphisms and AMD occurrence. However, we observed that AMD patients living in a rural environment and having the CC genotype of the IVS4+44C>A polymorphism had an increased risk of AMD, while individuals with the CA genotype or the A allele had a decreased risk of the disease. Moreover, in male AMD patients the C allele increased the risk of the disease, while the AA genotype decreased it.

**Conclusions:**

These results suggest that the VS4+44C>A polymorphism of the DMT1 gene may interact with place of living and gender to modulate the risk of AMD.

## Introduction

Age-related macular degeneration (AMD) is the primary cause of irreversible vision loss among the elderly, in developed countries [1]. AMD is ex definitio an age-related disease, and age is its strongest risk factor. It is estimated that about 30% of individuals aged 75 or more are diagnosed with AMD [2], and this proportion is predicted to be constantly increasing due to growing live expectancy [3]. Apart from age, in some populations women are reported to be at a higher risk of AMD [4]. Caucasian ethnicity, as well as bright iris color, seems to predispose to AMD [5, 6]. However, the impact of sunlight exposure on AMD development has not been unambiguously established [7, 8]. Tobacco smoking is frequently reported to be an important AMD risk factor [6, 9, 10]. Cardiovascular diseases and hypertension [11], obesity [12, 13], and high-fat diet [14] are proved to enhance the risk of AMD, while the association of the disease with cataract and cataract surgery needs further research [15, 16].

AMD is a progressive disease, developing in its late stage to one of two clinically distinct forms — dry or wet [17]. The dry form (geographic atrophy) is characterized by drusen formation and the presence of retinal depigmentation paths as signs of the degeneration of photoreceptors together with retinal epithelium [18]. The less prevalent wet form of AMD (exudative, neovascular) is associated with a majority of total blindness incidents. The progress of the wet form of the disease is faster, with apparent choroidal neovascularization leading to leakages and bleeding into the retina [18, 19]. A local inflammatory process appears, and a central disciform scar is formed. Photoreceptors and retinal pigment epithelium degenerate, leading to the loss of central vision [19].

The etiology of AMD is complex, and the mechanism of retinal cell death has not been fully understood. The eye is constantly exposed to short-wavelength light [20]. That causes a high rate of reactive oxygen species (ROS) generation within the eye, and this effect is augmented by high oxygen pressure in the retina [21], high rate of catabolic reactions in the inner segments of photoreceptors, and the presence of photoreactive compounds and polyunsaturated fatty acids (PUFA) in the retinal tissue [22–24]. Inflammatory processes associated with AMD may be an additional source of ROS [25].

The level of oxidative imbalance in the cell may exceed its oxidative defense capacity. Then it can cause oxidative damage to different cellular components including DNA, promoting apoptosis [26, 27]. It is proved that iron ions may generate free radicals in vivo by Fenton reaction, and that a number of disorders developes through the iron-dependent oxidative events [28, 29]. Severe iron overload leads to organ failure, and may cause neurodegeneration [28, 29].

Genetic factors are considered to play an important role in AMD, as has been shown in a number of family and twin studies [30]. Several genetic risk markers have been identified in this disease [23, 31]. It is thought that strong genotype–environment interaction appears in the AMD incidence [32]. In the present report, we show the interplay between the IVS4+44C>A polymorphism in the DMT1 gene and environmental factors in the AMD occurrence. The DMT1 gene (also known as SLC11A2, NRAMP2) encodes transmembrane transporter of iron and other divalent ions. It plays an important role in iron uptake, and participates in keeping iron homeostasis in the organism [33].

## Materials and methods

### Clinical subject

This study included a group of 436 individuals — 290 with the wet form of AMD, 148 with the dry form of the disease, and 168 controls. Medical history was obtained from all subjects, and no one reported any genetic disease. The patients underwent ophthalmic examination, including best-corrected visual acuity, intraocular pressure, slit-lamp examination, and fundus examination, performed with a slit lamp equipped with either non-contact or contact fundus lenses. The criteria for enrolling patients into the study groups were based primarily on clinical usefulness; the dry form group corresponded to AREDS categories 2, 3 and 4 (geographic atrophy subtype) and the wet to AREDS category 4 (choroidal neovascularisation or neovascular maculopathy subtype) [34]. Diagnosis of AMD was confirmed by optical coherence tomography (OCT) and, in some cases, by fluorescein angiography (FA) and indocyanin green angiography (ICG). OCT evaluated retinal thickness, the presence of RPE atrophy, drusen, or subretinal fluid and intraretinal edema; angiography assessed the anatomical status of the retinal vessels, the presence of choroidal neovascularization, and leakage. The OCT examinations were performed with Stratus OCT model 3000, software version 4.0 (Oberkochen, Germany). The FA and ICG examinations were completed with a Topcon TRC-50I IX fundus camera equipped with the digital Image Net image system, version 2.14 (Topcon, Tokyo, Japan). A structured questionnaire was used to get information from study subjects about lifestyle habits and family/personal history of AMD. The genetic analyses did not interfere with diagnostic or therapeutic procedures for the subjects. The Bioethics Committee of the Medical University of Warsaw, Poland approved the study, and each patient gave written informed consent.

### DNA isolation

The sample of whole venous blood was collected from every subject to EDTA-containing tubes. DNA was isolated from each sample using AxyPrep Blood Genomic DNA Miniprep kit (Axygen Biosciences, San Francisco, CA, USA) and stored deep frozen (−20°C) until use.

### Genotyping

#### PCR reaction

Each reaction tube contained 10 ng of genomic DNA, 0.75U Taq Polymerase (Biotools, Madrid, Spain), 1 × reaction buffer, 0.5 mM dNTP, 1.5 mM MgCl_2_ and 0.25 μM of each primer (Sigma-Aldrich, St. Louis, MO, USA). The sequence of primers for the IVS4+44C>A polymorphism was as in reference [35]. The sequences of primers, and length of PCR and restriction products for both polymorphisms, are given in Table 1. PCR was run on a Bio-Rad C1000^TM^ thermocycler (BIO-RAD Laboratories, Hercules, CA, USA) Thermal cycling conditions for the IVS4+44C>A polymorphism were: initial denaturation step at at 95°C for 3 min, 34 cycles of denaturation at 95°C for 30 s, annealing at 60°C for 30 s and amplification at 72°C for 1 min; final extension at 72°C for 5 min. For the c.2044T>C polymorphism, these conditions were: initial denaturation step at 95°C for 5 min, 33 cycles of denaturation at 95°C for 30 s, annealing at 62°C for 30 s and amplification at 72°C for 1 min; final extension at 72°C for 5 min.

**Table 1:** Sequences of primers and lengths of PCR and restriction products

Genotype/allele	Primer sequences and DNA fragments after digestion [bp]
IVS4 + 44	F: 5′ GACACATGCAATATCTGACATTG 3′
[352 bp]^a^	R: 5′ AGGCTACTATCCAACATGCAG 3′
CC	183, 100, 35, 34
CA	217, 183, 100, 35, 34
AA	217, 100, 35
Genotype/allele	Primer sequences and DNA fragments after digestion [bp]
c.2044T>C	F: 5′ AAATTTCTCAGCCTTTAAAAATCC3′
[231 bp]^a^	R: 5′ TTGAAAAGCTGACATTTGCTG 3′
TT	231
TC	231, 145, 86
CC	145, 86

F — forward primer, R — reverse primer, ^a)^ PCR product length

#### Enzyme reactions

Amplified DNA fragment containing IVS4+44C/A site was incubated with 1.5 U of MnlI restriction enzyme (AKOR Laboratories, Gdansk, Poland) for 4 h at 37°C, while fragment containing the c.2044T>C site was incubated with 1.5U of RsaI restrictase (Fermentas, Hanover, MD,USA) for 2.5 h in 37°C. The length of digestion products is shown in Table 1.

**Fig. 1:**
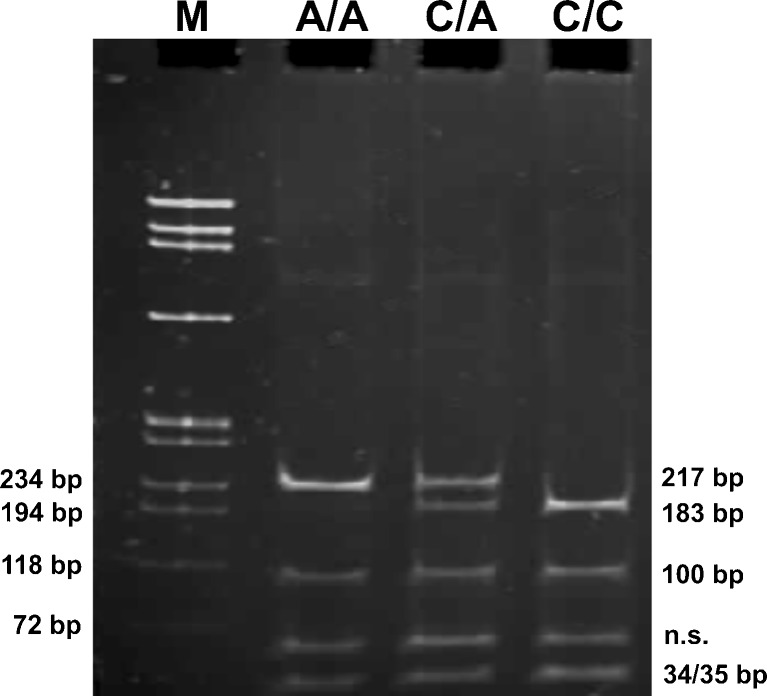
The frequent gel from the IVS4+44C>A polymorphism analysis. The first line (*M*) is a DNA ladder. Two non-specific bands were visible on all gels from IVS4+44C>A site analysis

**Fig. 2:**
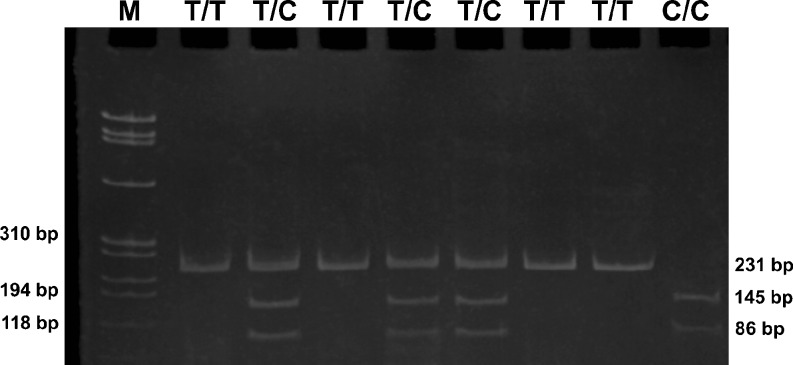
The frequent gel from the c.2044T>C polymorphism analysis. The first line (*M*) is a DNA ladder

After digestion samples were separated on 10% polyacrylamide gel, electrophoresis was run at 5 V/cm in BLUESTAR apparatus (DNA-Gdansk, Gdynia, Poland) in Tris-borate–EDTA buffer. ΦX-174 DNA/BsuRI (HaeIII) DNA ladder was utilized as a mass marker. After separation, gels were stained with ethidium bromide (0.5 μg/ml) and documented by the digital imaging system InGenius Bio Imaging (Syngene, Cambridge, UK). Representative gels for genotyping the IVS4+44C/A and c.2044T>C polymorphism are shown in Figs. 1 and 2 respectively.

### Data analysis

The allelic frequencies were calculated by gene counting, and genotypes were scored. The significance of the differences between distributions of genotypes and alleles was tested using the χ^2^ analysis. Unconditional logistic regression analysis was performed to assess the association between the genotypes of the polymorphisms and AMD incidence. The genotype-associated risk was expressed by crude odds ratio with 95% confidence intervals and the *p* value. Odds ratios were then adjusted for possible interfering factors. To verify a potential gene–environment interaction, the patients and controls were stratified depending on age, sex, living environment (rural or urban), smoking status and body mass index (BMI). Multiple unconditioned logistic regression analyses were run to test the association of genotypes and environmental and social factors with AMD occurrence. Statistical analysis was performed using the Statistica 9.0 package (Statsoft, Tulsa, OK, USA).

## Results

The frequencies of genotypes in the groups did not differ significantly from Hardy-Weinberg equilibrium — *p* > 0.05 for each group. The patient characteristics contained information about environmental and social factors having possible impact on AMD incidence. The analysis of the association between potential risk factors — age, sex, inhabitation, smoking habit, and Body Mass Index and AMD independently from genotypes was performed (Table 2). We found no association of sex, environment, BMI, and tobacco smoking with AMD. We observed a significant association of AMD with age (OR 1.04; 95% CI 1.02–1.06; *p* < 0.001; for every additional year) and the occurrence of the disease among 1st-degree relatives (OR 10.81; 95% CI 3.31–35.36; *p* < 0.001). The next step was to test the association between the genotypes of both polymorphisms and AMD. No association was found between the risk of AMD and the genotypes of that polymorphism in the group of all AMD patients (dry and wet forms) (Table 3.). Similarly, neither polymorphism correlated with the wet (Table 4) or dry (Table 5) forms of AMD. Then, the frequency of the complex genotypes of both polymorphic sites were counted (Table 6.). We found no association between these complex genotypes and AMD. Then, the patients were classified depending on potential risk factors, and tested separately. We found no associations in the case of the c.2044T>C polymorphisms. We did not observe any interaction of genotypes with age, sex, BMI, or tobacco smoking in AMD patients (data not shown). However, we found that the CC genotype of the IVS4 + 44C>A polymorphism was positively correlated with AMD (OR 3.50; 95% CI 1.19–10.31; *p* < 0.05), and the CA genotype and the A allele was inversely correlated with AMD (OR 0.29; 95% CI 0.10–0.84; *p* < 0.05) in the group of rural inhabitants (Table 7). No correlation was found in the group of industrial inhabitants. The AA allele of the IVS4+44C>A polymorphism was negatively correlated with AMD (OR 0.11; 95% CI 0.01–0.98; *p* < 0.05), while the C allele had a strong positive linkage with AMD among males (OR 9.56; 95% CI 1.02–89.99; *p* < 0.05) (Table 8).

**Table 2:** Association of AMD with age, sex, tobacco smoking, AMD in family, BMI, and living environment

Risk Factor	OR (95% CI)^1^
Age (for +1 year)	1.04 (1.02–1.06); *p* < 0.001^2^
Sex (for females)	0.68 (0.4–1.02)
Tobacco smoking (never vs ever)	0.82 (0.55–1.23)
AMD among 1st-degree relatives	10.81 (3.31–35.36); *p* < 0.001^2^
Body Mass Index (for +1 BMI unit)	0.97 (0.92–1.02)
Environment (for countryside)	0.74 (0.43–1.28)

^1^Odds ratio with 95% confidence interval, ^2^ Chi-square test

**Table 3:** Distribution of genotypes, frequency of alleles of the IVS4+44C>A and c.2044T>C polymorphism of the *DMT1* gene, and odds ratios (OR) with 95% confidence intervals (95% CI) in age-related macular degeneration and controls

Genotype/allele	Control (158)	AMD (381)	^A^ OR (95% CI)	^B^ OR^adjusted^ (95% CI)
IVS4+44C>A	*N* (%)	*N* (%)		
CC	109 (0.69)	262 (0.69)	0.99 (0.66–1.48)	1.43 (0.79–2.60)
CA	46 (0.29)	109 (0.29)	0.98 (65–1.47)	0.76 (0.41–1.38)
AA	3 (0.02)	10 (0.03)	1.39 (0.38–51.3)	0.38 (0.06–2.51)
C	264 (0.84)	633 (0.83)	0.97 (0.68–1.38)	2.63 (0.40–17.43)
A	52 (0.16)	129 (0.17)	1.03 (0.73–1.47)	0.73 (0.41–1.32)
Genotype/allele	Control (168)	AMD (436)	^A^ OR (95% CI)	^B^ OR^adjusted^ (95% CI)
c.2044T>C	N (%)	N (%)		

TT	127 (0.76)	320 (0.73)	0.86 (0.59–1.34)	1.23 (0.65–2.35)
TC	39 (0.23)	108 (0.25)	1.09 (0.72–1.66)	0.79 (0.41–1.53)
CC	2 (0.01)	8 (0.02)	1.55 (0.33–7.38)	1.75 (0.19–15.86)
T	293 (0.87)	748 (0.86)	0.86 (0.59–1.26)	0.87 (0.09–8.37)
C	41 (0.12)	122 (0.14)	1.17 (0.80–1.70)	0.77 (0.40–1.47)

^A^Crude odds ratio with 95% confidence interval; ^B^ Odds ratio adjusted for age, sex, and environment of living

**Table 4:** Distribution of genotypes, frequency of alleles of the IVS4+44C>A and c.2044T>C polymorphism of the *DMT1* gene, and odds ratios (OR) with 95% confidence intervals (95% CI) in wet form of age-related macular degeneration and controls

Genotype/allele	Control (158)	Wet AMD (233)	^A^ OR (95% CI)	^B^ OR^adjusted^ (95% CI)
IVS4+44C>A	*N* (%)	*N* (%)		
CC	109 (0.69)	163 (0.70)	1.13 (0.72–1.76)	1.57 (0.79–3.12)
CA	46 (0.29)	65 (0.28)	0.94 (0.60–1.47)	0.73 (0.37–1.45)
AA	3 (0.02)	5 (0.02)	0.66 (0.16–2.80)	0.16 (0.01–1.95)
C	264 (0.84)	391 (0.84)	1.03 (0.70–1.51)	6.29 (0.51–77.15)
A	52 (0.16)	75 (0.16)	0.97 (0.66–1.43)	0.69 (0.35–1.36)
Genotype/allele	Control (168)	Wet AMD (290)	^A^ OR (95% CI)	^B^ OR^adjusted^ (95% CI)
c.2044T>C	*N* (%)	*N* (%)		

TT	127 (0.76)	217 (0.75)	0.96 (0.62–1.49)	1.30 (0.62–2.70)
TC	39 (0.23)	70 (0.24)	1.05 (0.67–1.65)	0.82 (0.39–1.71)
CC	2 (0.01)	3 (0.01)	0.87 (0.14–5.25)	1.23 (0.10–15.21)
T	293 (0.87)	504 (0.87)	0.93 (0.62–1.39)	2.62 (0.15–45.54)
C	41 (0.12)	76 (0.13)	1.08 (0.72–1.62)	0.70 (0.33–1.47)

^A^ Crude odds ratio with 95% confidence interval; ^B^ Odds ratio adjusted for age, sex and environment of living

**Table 5:** Distribution of genotypes, frequency of alleles of the IVS4+44C>A and c.2044T>C polymorphism of the *DMT1* gene, and odds ratios (OR) with 95% confidence intervals (95% CI) in dry form of age-related macular degeneration and controls

Genotype/allele	Control (158)	Dry AMD (148)	^A^ OR (95% CI)	^B^ OR^adjusted^ (95% CI)
IVS4+44C>A	*N* (%)	*N* (%)		
CC	109 (0.69)	99 (0.67)	0.91 (0.56–1.47)	1.27 (0.62–2.60)
CA	46 (0.29)	44 (0.30)	1.03 (0.63–1.68)	0.82 (0.40–1.69)
AA	3 (0.02)	5 (0.03)	1.81 (0.42–7.70)	0.65 (0.08–5.19)
C	264 (0.84)	242 (0.82)	0.88 (0.58–1.34)	1.54 (0.19–12.28)
A	52 (0.16)	54 (0.18)	1.13 (0.75–1.72)	0.79 (0.38–1.61)
Genotype/allele	Control (168)	Dry AMD (146)	^A^ OR (95% CI)	^B^ OR^adjusted^ (95% CI)
c.2044T>C	*N* (%)	*N* (%)		

TT	127 (0.76)	103 (0.71)	0.77 (0.47 - 1.28)	1.14 (0.53–2.48)
TC	39 (0.23)	38 (0.26)	1.16 (0.70 - 1.95)	0.78 (0.35–1.73)
CC	2 (0.01)	5 (0.03)	2.94 (0.56 - 15.40)	2.31 (0.22–24 .03)
T	293 (0.87)	244 (0.84)	0.71 (0.45 - 1.12)	0.43 (0.04–4.52)
C	41 (0.12)	48 (0.16)	1.41 (0.90 - 2.21)	0.88 (0.40–1.90)

^A^ Crude odds ratio with 95% confidence interval; ^B^ Odds ratio adjusted for age, sex and environment of living

**Table 6:** Distribution of combined genotypes of the IVS4+44C>A and c.2044T>C polymorphism of the *DMT1* gene and odds ratios (OR) with 95% confidence intervals (95% CI) in age-related macular degeneration and controls

Genotype	Control (158)	AMD (377)	^A^ OR (95% CI)	^B^ OR^adjusted^ (95% CI)
IVS4+44C>A /c.2044T>C	*N* (%)	*N* (%)		
CC/TT	107 (0.68)	249 (0.66)	0.93 (0.62–1.38)	1.33 (0.74–2.38)
CC/TC	2 (0.01)	8 (0.02)	1.69 (0.36–8.05)	2.19 (0.19–25.36)
CC/CC	0 (0)	1 (0)	–	–
CA/TT	12 (0.08)	25 (0.07)	0.86 (0.42–1.77)	0.73 (0.28–1.96)
CA/TC	34 (0.22)	84 (0.22)	1.05 (0.67–1.64)	0.81 (0.41–1.59)
CA/CC	0 (0)	0 (0)	–	–
AA/TT	0 (0)	0 (0)	–	–
AA/TC	1 (0.01)	4 (0.01)	1.68 (0.19–15.18)	–
AA/CC	2 (0.01)	6 (0.02)	1.26 (0.25–6.32)	0.91 (0.09–9.32)

^A^ Crude odds ratio with 95% confidence interval; ^B^ Odds ratio adjusted for age. sex and environment of living

**Table 7:** Distribution of genotypes. frequency of alleles of the IVS4+44C>A and c.2044T>C polymorphism of the *DMT1* gene, and odds ratios (OR) with 95% confidence intervals (95% CI) in age-related macular degeneration and controls among urban and countryside ancestors

	Urban district			Countryside		
Genotype/allele	Control (55)	Dry AMD (113)	^A^ OR (95% CI)	Control (33)	Dry AMD (50)	^A^ OR (95% CI)
IVS4+44C>A	*N* (%)	*N* (%)		*N* (%)	*N* (%)	
CC	40 (0.73)	77 (0.68)	0.94 (0.45–1.99)	20 (0.61)	40 (0.80)	3.50 (1.19–10.31) *
CA	13 (0.24)	33 (0.29)	1.24 (0.58–2.68)	13 (0.39)	10 (0.20)	0.29 (0.10–0.84) *
AA	2 (0.04)	3 (0.03)	0.31 (0.04–2.18)	0 (0)	0 (0)	–
C	93 (0.85)	187 (0.83)	3.25 (0.46–23.04)	53 (0.80)	90 (0.90)	–
A	17 (0.15)	39 (0.17)	1.14 (0.54–2.39)	13 (0.20)	10 (0.10)	0.29 (0.10–0.84) *
Genotype/allele	Control (56)	Dry AMD (113)	^A^ OR (95% CI)	Control (33)	Dry AMD (50)	^A^ OR (95% CI)
c.2044T>C	*N* (%)	*N* (%)		*N* (%)	*N* (%)	

TT	45 (0.80)	82 (0.73)	0.789 (0.35–1.78)	23 (0.70)	42 (0.84)	3.03 (0.94–9.76)
TC	10 (0.18)	27 (0.24)	1.29 (0.56–3.00)	10 (0.30)	7 (0.14)	0.33 (0.10–1.07)
CC	1 (0.02)	4 (0.03)	0.97 (0.10–9.77)	0 (0)	1 (0.02)	–
T	100 (0.89)	191 (0.85)	1.03 (0.10–10.30)	56 (0.85)	91 (0.91)	–
C	12 (0.11)	35 (0.15)	1.19 (0.52–2.69)	10 (0.15)	9 (0.09)	0.33 (0.10–1.07)

^A^ Odds ratio adjusted for age and sex; * *p* < 0.05

**Table 8:** Distribution of genotypes. frequency of alleles of the IVS4+44C>A and c.2044T>C polymorphism of the *DMT1* gene, and odds ratios (OR) with 95% confidence intervals (95% CI) in age-related macular degeneration and controls among males and females

	Males			Females		
Genotype/allele	Control (39)	Dry AMD (127)	^A^ OR (95% CI)	Control (119)	Dry AMD (254)	^A^ OR (95% CI)
IVS4+44C> A	*N* (%)	*N* (%)		*N* (%)	*N* (%)	
CC	27 (0.69)	78 (0.61)	1.49 (0.49–4.57)	82 (0.69)	184 (0.72)	1.44 (0.71–2.93)
CA	10 (0.26)	44 (0.35)	1.09 (0.34–3.47)	36 (0.30)	65 (0.26)	0.66 (0.32–1.35)
AA	2 (0.05)	5 (0.04)	0.11 (0.01–.98) *	1 (0.01)	5 (0.02)	–
C	64 (0.82)	200 (0.79)	9.56 (1.02–9.99) *	200 (0.85)	433 (0.85)	–
A	14 (0.18)	54 (0.21)	0.67 (0.22–2.05)	38 (0.16)	75 (0.15)	0.75 (0.37–1.51)
Genotype/allele	Control (43)	Dry AMD (129)	^A^ OR (95% CI)	Control (125)	Dry AMD (306)	^A^ OR (95% CI)
c.2044T>C	*N* (%)	*N* (%)		*N* (%)	*N* (%)	

TT	32 (0.74)	87 (0.67)	2.00 (0.60–6.66)	95 (0.76)	232 (0.76)	1.04 (0.48–2.27)
TC	10 (0.23)	37 (0.29)	0.57 (0.17–1.94)	29 (0.23)	71 (0.23)	0.91 (0.42–1.99)
CC	1 (0.03)	5 90.04)	0.45 (0.04–5.13)	1 (0.01)	3 (0.01)	–
T	74 (0.86)	211 (0.82)	2.24 (0.20–25.72)	219 (0.88)	535 (0.87)	–
C	12 (0.14)	47 (0.18)	0.47 (0.14–1.58)	31 (0.12)	77 (0.13)	0.92 (0.42–2.02)

^A^ Odds ratio adjusted for age and living environment; * p < 0.05

## Discussion

Age-related macular degeneration is an important health problem in developed countries. A constantly growing percentage of affected individuals and the estimated tendency that it will rise over the next years is an argument confirming the urgency of revealing the nature of AMD. That includes searching for genetic markers of this disease, since it is established that AMD have features of inherited disease [30–32]. Several genetic polymorphisms significantly affecting AMD has been identified. These include polymorphisms in the CFH, CFB, C2, ERCC6, HTRA, VEGF and other genes [36]. In the present study, we analyzed polymorphisms in the divalent metal transporter 1 (DMT1) gene and their association with AMD risk. Iron homeostasis is crucial for proper functioning of the organism, and while iron deficiency leads to anemia, its excess may cause severe symptoms. In a number of cases, iron overload leads to heart, liver or brain damage [37, 38]. It is particularly visible in hereditary haemochromatosis — genetic defects in one of the iron homeostasis genes, associated with iron accumulation in the organism [39]. Moreover, the elevated iron level may stimulate cancer transformation [40]. Many age-related diseases may be connected with an elevated iron level associated with aging [41, 42]. The mechanism of iron-related harmful effects in the organism involves the action of free radicals. Free divalent iron ions participate in the Fenton reaction, producing highly reactive hydroxyl radicals, which may damage cellular components, including DNA, and impair DNA repair [43]. An elevated level of chelatable iron ions has been observed in maculas derived post mortem from AMD patients [44]. Mice lacking key genes of iron homeostasis – ceruloplasmin, hephaestin and hepcidin — developed retinal degeneration with features of AMD [45, 46]. Similarly, the case of a patient with a defect in the ceruloplasmin gene has been reported. The patient suffered from AMD, and an increased iron content in the macula of the patient was shown [47]. It was also shown, in a mouse model of AMD and RPE cell line, that iron chelation protected from AMD development [45, 48].

In this paper, we have analyzed two SNP polymorphisms in the DMT1 gene. This gene has two alternative promoters and two alternative polyadenylation sites. Four main isoforms of DMT1 have been identified. Depending on the choice of polyadenylation site, the transcript may include the iron response element (IRE) in the 3’UTR region [49, 50]. The differences between isoforms determine mainly the tissue-specific expression pattern of the final protein [51]. DMT1 plays a role in iron absorption from the intestine and its endosomal transport in the cell, being responsible for cellular distribution of this metal [52, 53]. DMT1 also plays an important role in cation balance in the nervous system [52, 53]. There are reports concerning imbalance of iron in the organism as a consequence of mutations in the DMT1 gene [54, 55]. It has also been postulated that DMT1 plays a role in the process of neurodegeneration [56]. The IVS4+44C>A polymorphism is located in the intron 4 of the DMT1 gene. A nucleotide change inside the intron may affect significantly either constitutive splicing or alternative splicing by the corruption of splicing regulatory *cis*-elements, giving incorrect isoforms of the protein [57, 58]. This polymorphism has been reported not to be associated with inflammatory bowel disease [59] and hereditary haemochromatosis [60], but its association with Parkinson’s disease has been shown [35]. We did not find any report on the functional significance of the other polymorphism, c.2044T>C.

Our genotype-independent analysis of potential AMD risk factors showed an association with age and a strong association with familial AMD. Therefore, positive AMD familial history may increase the risk of the occurrence of the disease among remaining family members. On the other hand, we found no association of AMD with smoking status, sex, BMI, and place of living. In particular, the lack of association of AMD with sex and smoking is somehow surprising, because several groups have shown a significant association between tobacco smoking and AMD [61]. Furthermore, female sex was reported as an AMD risk factor in several populations [4]. We do not have information on the general association between AMD occurrence and gender in Poland, so we can conclude that the dependence of the AMD risk on sex may be population-specific, or that our studies lacked power to detect this dependence, and further investigations in the Polish population are needed to clarify this point. Tobacco smoking is well-confirmed as a modulator of AMD risk, although some groups have reported no association between smoking and AMD, or only a limited, borderline association [62–65]. Again, the dependence between smoking and AMD may be population-specific and influenced by passive smoking, which should not be ignored [66]. Stratification of patients depending on age, sex, BMI, living environment and tobacco smoking status revealed no correlation between these factors, AMD, and the genotypes of the c.2044T>C polymorphism. However, in the group of rural inhabitants, the polymorphism IVS4+44C>A was significantly correlated with AMD risk. The C variant seemed to increase the AMD risk if it occurred among rural inhabitants, while the A variant had a protective role. Similarly, the C variant strongly positively increased AMD risk among males, and the A variant decreased the risk in this group. This is particularly interesting, since in a number of researches in other populations, females were at higher risk of AMD [4]. No significant influence of a tobacco smoking habit is also worth emphasizing, as tobacco smoking is thought to be a major risk factor in AMD. Our result showed that the IVS4+44C>A polymorphism in DMT1 gene may be considered as a potential environment-dependent risk marker for AMD.
